# EUS-Guided Versus Percutaneous Celiac Neurolysis for the Management of Intractable Pain Due to Unresectable Pancreatic Cancer: A Randomized Clinical Trial

**DOI:** 10.3390/jcm9061666

**Published:** 2020-06-01

**Authors:** Won Jae Yoon, Yul Oh, Changhoon Yoo, Sunguk Jang, Seong-Sik Cho, Jeong-Hun Suh, Seong-Soo Choi, Do Hyun Park

**Affiliations:** 1 Department of Internal Medicine, College of Medicine, Ewha Womans University, Seoul 07804, Korea; biliary@naver.com; 2 Department of Anesthesiology and Pain Medicine, Asan Medical Center, University of Ulsan College of Medicine, Seoul 05505, Korea; dhdbf@hanmail.net (Y.O.); paindrsuh@gmail.com (J.-H.S.); 3 Department of Oncology, Asan Medical Center, University of Ulsan College of Medicine, Seoul 05505, Korea; gooddac@gmail.com; 4 Department of Gastroenterology and Hepatology, Cleveland Clinic, Cleveland, OH 44195, USA; jangs@ccf.org; 5 Department of Occupational and Environmental Medicine, College of Medicine, Dong-A University, Busan 49201, Korea; 0361pt@hanmail.net; 6 Division of Gastroenterology, Department of Internal Medicine, Asan Medical Center, University of Ulsan College of Medicine, Seoul 05505, Korea

**Keywords:** pancreatic cancer, pain, celiac neurolysis, endoscopic ultrasound

## Abstract

Although endoscopic ultrasound-guided celiac neurolysis (EUS-CN) and percutaneous celiac neurolysis (PCN) are utilized to manage intractable pain in pancreatic cancer patients, no direct comparison has been made between the two methods. We compared the efficacy and safety of EUS-CN and PCN in managing intractable pain in such patients. Sixty pancreatic cancer patients with intractable pain were randomly assigned to EUS-CN (*n* = 30) or PCN (*n* = 30). The primary outcomes were pain reduction in numerical rating scale (NRS) and opioid requirement reduction. Secondary outcomes were: successful pain response (NRS decrease ≥50% or ≥3-point reduction from baseline); quality of life; patient satisfaction; adverse events; and survival rate at 3 months postintervention. Both groups reported sustained decreases in pain scores up to 3 months postintervention (mean reductions in abdominal pain: 0.9 (95% confidence interval (CI): −0.8 to 4.2) and 1.7 (95% CI: −0.3 to 2.1); back pain: 1.3 (95% CI: −0.9 to 3.4) and 2.5 (95% CI: −0.2 to 5.2) in EUS-CN, and PCN groups, respectively). The differences in mean pain scores between the two groups at baseline and 3 months were −0.5 (*p* = 0.46) and −1.4 (*p* = 0.11) for abdominal pain and 0.1 (*p* = 0.85) and −0.9 (*p* = 0.31) for back pain in favor of PCN. No significant differences were noted in opioid requirement reduction and other outcomes. EUS-CN and PCN were similarly effective and safe in managing intractable pain in pancreatic cancer patients. Either methods may be used depending on the resources and expertise of each institution.

## 1. Introduction

Pancreatic cancer has an overall 5-year survival rate of about 6% and is thus one of the leading contributors of cancer-related deaths in the world [1]. Whereas surgical resection is regarded to offer the only chance of cure, more than 80% of patients are deemed ineligible for resection [2,3]. In such patients with unresectable pancreatic cancer, intractable pain is the most common yet important symptom that significantly degrades their quality of life (QOL) [4,5,6]. Abdominal pain is the most common reason for emergency room visits in patients with pancreatic cancer during the final six months of their lives [7]. As the pain in pancreatic cancer primarily involves neuropathic, visceral, and somatic mechanisms [8], opioids are given to those with moderate-to-severe degrees of pain [9]. However, patients’ responses to opioids are variable and dissipate over time. Moreover, the side effects of opioids such as nausea, vomiting, dry mouth, constipation, and drowsiness further reduce the QOL, and thus preclude adequate dosing [5,10]. Therefore, in such cases of patients with intractable pain due to pancreatic cancer, alternative approaches such as celiac neurolysis (CN) are used.

A commonly used method of CN is percutaneous CN (PCN) [5,10], which can be done under the guidance of fluoroscopy, computed tomography (CT), or ultrasound [11]. Ethanol of varying concentrations ranging from 50% to 100% is the preferred neurolytic agent in PCN [12]. PCN has been shown to be effective in reducing pain [13], opioid use, and opioid-induced side effects in patients with abdominal cancers compared with systemic analgesic therapy [12]. 

CN can also be carried out under endoscopic ultrasound (EUS) guidance. Two methods of EUS-guided CN (EUS-CN) are available: EUS-guided celiac plexus neurolysis (EUS-CPN), which injects neurolytic agents at or near the plexus of celiac nerve; and EUS-guided celiac ganglia neurolysis, which directly injects neurolytic agents in the celiac ganglia [14,15,16]. EUS-CN is preferred over PCN in some institutions with expertise [16,17], as it also confers better pain relief compared with systemic analgesic therapy [18] and potentially improved safety by using a transgastric approach, which allows direct access to the celiac plexus to reduce the risk of injuries to the spinal nerve, diaphragm, or spinal artery [5,14,19]. 

Although both EUS-CN and PCN are widely used, the selection between the two methods has not been based on robust evidence, let alone a randomized, controlled study comparing EUS-CN and PCN for management of cancer pain. One recent expert panel-based guideline [20] recommended EUS-CN over PCN for celiac plexus ablation, but only by citing a paper [21] published in 1999 that targeted chronic pancreatitis rather than pancreatic cancer. This randomized trial aimed to compare the efficacy and safety of EUS-CN and PCN in managing intractable pain in patients with pancreatic cancer. The two methods were compared in terms of reduction of pain and opioid requirement, QOL, patient satisfaction, adverse events (AEs), and survival rate at 3 months after intervention.

## 2. Patients and Methods

### 2.1. Study Design and Participants

This prospective, randomized, assessor blind study was conducted at Asan Medical Center in Seoul, Korea and was registered in Clinical Research Information Service (KCT0002350). The study was approved by the Institutional Review Board of Asan Medical Center (approval number: 2017-0186). We followed the CONSORT guidelines to report this study. Patients with pancreatic cancer who were deemed as non-surgical candidates and also met the following criteria were considered for randomization: (1) diagnosis of pancreatic cancer based on clinical, radiological, or pathological assessment; (2) referred for abdominal and/or back pain due to pancreatic cancer; (3) between 20 and 80 years of age; (4) no prior CN; (5) cancer pain unresponsive to the WHO 3-step analgesic ladder; and (6) willingness to consent for the participation in the trial. Patients were excluded if they: (1) did not agree to participate in the study; or had (2) surgically resectable pancreatic cancer; (3) documented side effects to local anesthetics or steroids; (4) pain unrelated to pancreatic cancer; (5) hemostatic abnormality; (6) evidence of concurrent infection; (7) yellow flag sign [22]; or (8) red flag sign [22]. All participants provided written informed consent. This study was conducted in accordance with the Declaration of Helsinki. All authors had access to the study data and reviewed and approved the final manuscript.

### 2.2. Randomization and Masking

Patients who met the inclusion criteria were allocated in a 1:1 ratio to EUS-CN or PCN by randomization without risk stratification. Block randomization was employed in order to assign equal numbers of patients in each treatment group; block sizes were randomly permuted to make the allocation process unpredictable. Randomization was conducted by using a web-based program (http://www.randomization.com) by a clinical research coordinator who was not involved in patient diagnosis. The patients and the attending physicians (i.e., endoscopists and anesthesiologists) were not blinded to the treatment allocation; the physicians who performed the procedures were not actively involved in the care of the participants. The doses of opioids were adjusted by the attending medical oncologists to provide best supportive care for the patients. Both the assessor (Y.O.) of the patients after the intervention and the statistician (S.Si.C.) who analyzed the data were blinded to the treatment allocation. 

### 2.3. EUS-CN

**Figure 1:**
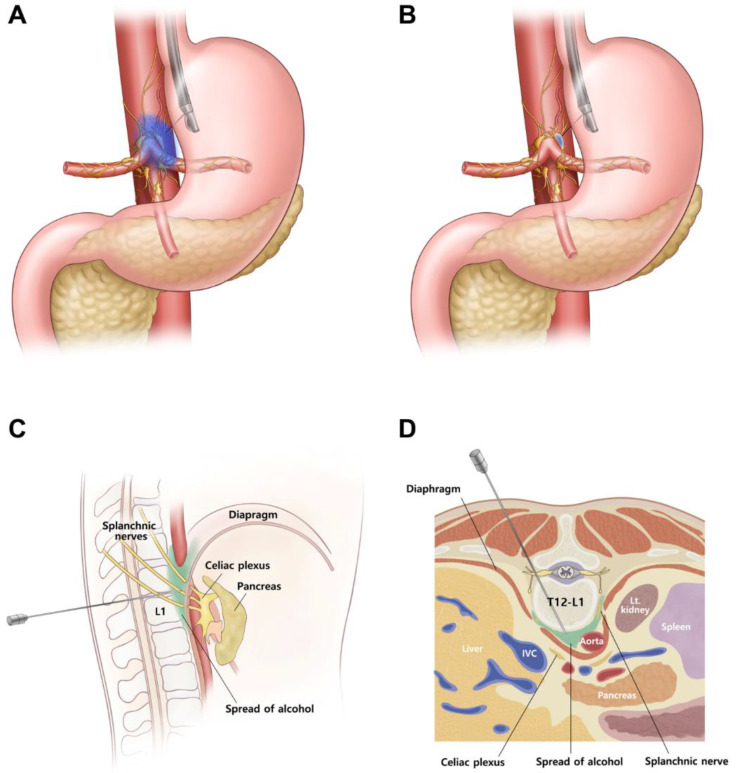
Schematic diagram of endoscopic ultrasound (EUS)-guided and percutaneous transdiscal celiac neurolysis. (**A**), EUS-guided celiac plexus neurolysis. (**B**), EUS-guided celiac ganglion neurolysis. (**C**), Sagittal view of percutaneous transdiscal celiac neurolysis. (**D**), Axial view of percutaneous transdiscal celiac neurolysis. Note that the needle is located at the retrocrural space. IVC, inferior vena cava; Lt. kidney, left kidney.

EUS-CNs were carried out on an inpatient basis. The attending physicians identified the celiac ganglia, which were typically found between the celiac artery and the left adrenal gland as hypoechoic nodular structures with thread-like hyperechoic structures [16]. If multiple ganglia were identified, the largest one was targeted for intervention. However, if multiple ganglia with a long diameter >12 mm were present, then neurolysis was considered for each ganglion [23]. EUS-guided celiac ganglion neurolysis plus unilateral (i.e., single central injection) CPN was performed on the celiac ganglia; if the celiac ganglia were not found, unilateral CPN was performed. The method for EUS-CN is described in detail in Supplementary File, and the schematic diagram of EUS-CN is presented in Figure 1A,B.

### 2.4. Percutaneous CN (PCN)

A fluoroscopy-guided transdiscal approach was used for PCNs [24]. Before the procedure, simulation for the transdiscal needle pathway was carried out using the most recent abdominal CT image [25]. The needle was introduced and advanced through the T12–L1 disc in accordance with the CT-simulated pathway. Immediately after penetrating the disc, PCN was performed after confirmation of the proper contrast dye spread pattern. The methods for PCN is described in detail in Supplementary File, and the schematic diagram of PCN is presented in Figure 1C,D.

### 2.5. Outcomes

The primary outcomes were the reduction in the severity of pain at 3 months after intervention and the changes in concomitant analgesic therapy. Severity of pain was assessed using a standardized numerical rating scale (NRS) ranging from 0 (no pain) to 10 (worst pain possible). Changes in concomitant analgesic therapy were evaluated by calculating the changes of daily opioid use converted to total daily oral morphine equivalent dose (MED), which assumes that different opioids with different doses produce a similar analgesic effect [26]. Secondary outcomes were proportions of successful pain response, QOL, patient satisfaction, incidence of AEs, and survival at 3 months after intervention. Successful pain response was defined as a decrease in NRS ≥50% relative to baseline or a ≥3-point reduction [17]. The National Comprehensive Cancer Network Functional Assessment of Cancer Therapy Hepatobiliary-Pancreatic Symptom Index (NFHSI) was used to measure the changes in QOL [27]. Participant satisfaction regarding the intervention was measured by the global perceived effect of satisfaction (GPES) using a 7-point Likert scale: the GPES takes into account all components of the participant’s experience such as pain relief, improvement in physical and emotional functioning, side effects, and convenience [28]. The incidence of AEs (i.e., procedure-related pain, diarrhea, and hypotension) was also calculated. All outcomes were assessed before the intervention (baseline) and at 1 week, 2 weeks, 1 month, 2 months, and 3 months after the intervention during outpatient visits or via phone calls. All measurements of baseline and postprocedural outcomes were performed by an independent physician (Y.O.) who was blinded to the treatment allocations.

### 2.6. Statistical Analysis

The number of participants was determined using the reduction in the severity of pain at 3 months after the intervention as measured with NRS. In previous studies, the difference in average NRS at 3 months after the CN procedure was 4.28 with a standard deviation of 0.68 in patients treated with EUS-CPN [12], and 3.69 with a standard deviation of 0.61 in patients with percutaneous fluoroscopy-guided transdiscal CPN [29]. To calculate sample size for an equivalence trial, we used the G*Power Version 3.1.7 (Kiel University, Kiel, Germany) to set the power and the probability of type I error at 0.9 and 0.05, respectively, and estimated effect size of 0.92 between two groups at 3 months after intervention using a two-sided Student t-test. As a result, at least 26 participants were required for each group. Estimating a dropout rate of 15%, a total of 60 patients were enrolled and equally allocated to each group. 

All observed data were analyzed on an intention-to-treat (ITT) basis, regardless of loss to follow-up or dropout from the study. Considering possible data loss resulting from dropout and treatment failure, a linear mixed-effect model was used to analyze and compare the continuous variables (NRS, MED, NFHSI, and GPES) between baseline and each follow-up. To compare the repeated data of successful responders (binary outcome) among groups, a generalized estimating equation was used. Patient survival at 3 months was estimated using the Kaplan–Meier method and compared using the log-rank test. Two-tailed *p* values < 0.05 were considered to indicate statistically significant difference. All data manipulations and statistical analyses except for survival analysis were performed using SPSS version 21 (IBM Corporation, Armonk, NY, USA) and Stata version 13.1 (StataCorp LP, College Station, TX, USA). Survival analyses were performed using MedCalc for Windows, version 15.0 (MedCalc Software, Ostend, Belgium).

## 3. Results

### 3.1. Patient Characteristics

**Figure 2:**
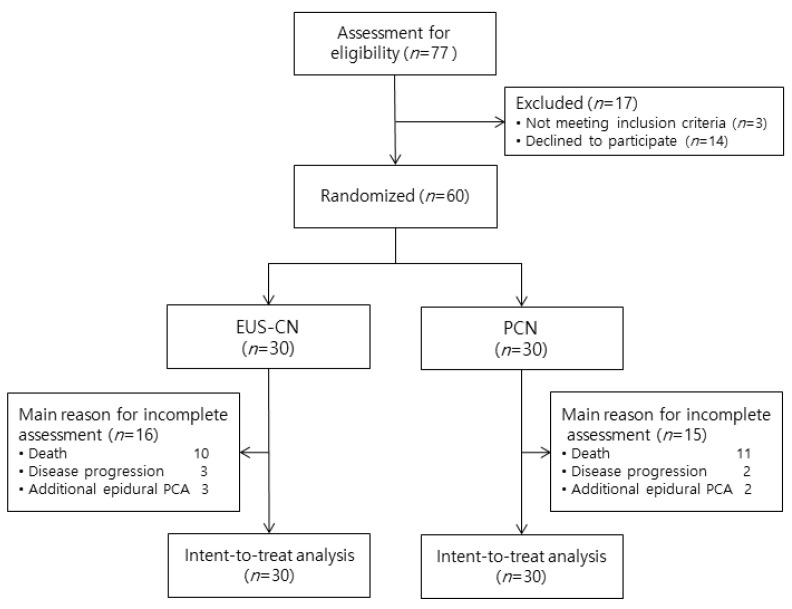
Patient flow diagram. EUS-CN, endoscopic ultrasound-guided celiac neurolysis; PCN, percutaneous celiac neurolysis; PCA, patient-controlled analgesia.

Between March 2017 and August 2018, 77 patients diagnosed with unresectable pancreatic cancer presenting with intractable pain were screened for eligibility to participate in the study. Seventeen patients were excluded (failure to meet the inclusion criteria: *n* = 3; declined to participate: *n* = 14). A total of 60 patients who fulfilled the inclusion criteria agreed to participate in this study. Thirty patients were randomized to each group. All participants received the allocated treatment and were included in the ITT population (Figure 2). 

The two groups did not show significant differences in the baseline demographic and clinical characteristics (Table 1). Except for five patients (two in the EUS-CN group and three in the PCN group), the study procedures were performed after progression on at least one line of chemotherapy, and 14 (46.6%) patients in the EUS-CN group and 11 (36.7%) patients in the PCN group underwent study procedures after progression on 2nd or greater line of chemotherapy. As the first-line therapy, gemcitabine plus nab-paclitaxel was the most commonly used regimen (*n* = 15, (50.0%) for EUS-CN group; *n* = 17 (56.7%) for PCN group). There were no significant differences in the baseline characteristics of the type and amount of the opioids (Supplementary Table S1). The mean injected volumes of local anesthetic in the EUS-CN group and the PCN group were 25.5 ± 7.7 mL and 9.5 ± 1.1 mL, respectively. The mean injected volumes of alcohol in the EUS-CN group and the PCN group were 36.8 ± 10.1 mL and 9.3 ± 1.3 mL, respectively. The mean procedure times of EUS-CN and PCN for 12.7 ± 3.8 min and 15.6 ± 5.4 min, respectively (*p* = 0.018).

### 3.2. Primary Outcomes

The estimated mean changes in pain scores are shown in Table 2 and Supplementary Figure S1. ITT analyses showed that the pain scores in the abdomens and backs of both groups had reduced at 3 months following each procedure. Specifically, for abdominal pain, the mean reductions in pain scores at 3 months were 0.9 (95% confidence interval (CI): −0.8 to 4.2) and 1.7 (95% CI: −0.3 to 2.1) in the EUS-CN and PCN groups, respectively; for back pain, the mean reductions in pain scores were 1.3 (95% CI: −0.9 to 3.4) and 2.5 (95% CI: −0.2 to 5.2) in the EUS-CN and PCN groups, respectively. 

For abdominal pain, the estimated differences of mean pain scores between the two groups were −0.5 (95% CI: −1.8 to 0.8) at baseline and −1.4 (95% CI: −3.1 to 0.3) at 3 months (*p* = 0.11) in favor of PCN, albeit no significant differences were observed between the two groups at each follow-up time point (Table 2). Similarly, the two groups did not show significant differences in back pain scores at each follow-up time point. 

**Figure 3:**
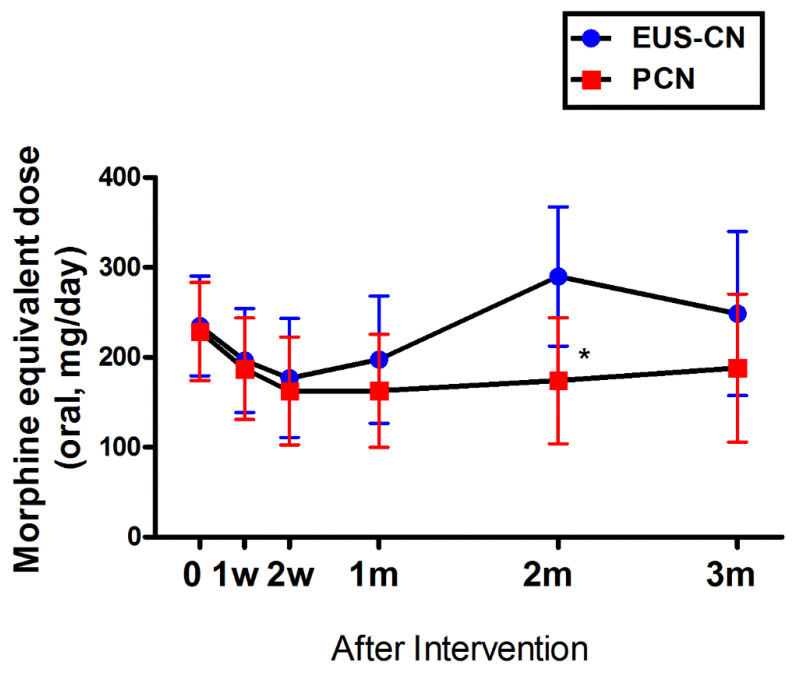
Changes in opioid consumption after EUS-CN or PCN. Opioid consumption was measured by calculating the daily opioid use converted to total daily oral morphine equivalent dose (MED). Adjusted prediction (95% CI) of opioid consumption in MED after intervention. A linear mixed model was used for the statistical analysis. Overall *p*-value between two groups for opioid consumption and difference from baseline was 0.22. CI, confidence interval; EUS-CN, endoscopic ultrasound-guided celiac neurolysis; PCN, percutaneous celiac neurolysis. * *p* < 0.05.

The overall difference in opioid consumption between the two groups was not statistically significant (*p* = 0.22), except at 2 months after intervention (Figure 3). Similarly, the overall estimated percent difference in opioid consumption from baseline after intervention between the two groups was not significantly significant (*p* = 0.17).

### 3.3. Secondary Outcomes

The estimated successful pain response rates were not significantly different between the two groups at 1 week (60.0%, 95% CI: 42.5 to 77.5) for EUS-CN vs. 56.7% (95% CI: 38.9 to 74.4) for PCN, *p* = 0.80), 1 month (56.7 %, 95% CI: 38.9 to 74.4) for EUS-CN vs. 70.0% (95% CI: 53.6 to 86.4) for PCN, *p* = 0.29), and 3 months (23.3%, 95% CI: 8.2 to 38.5) for EUS-CN and 36.7% (95% CI: 19.4 to 53.9) for PCN, *p* = 0.26) after treatment, respectively. The overall estimated proportions of successful pain response were not significantly different between the two groups (*p* = 0.33; Table 3). The QOL of both groups improved at 3 months following each procedure (Supplementary Figure S2 and Table S2), without significant differences between the two groups (*p* = 0.44). As shown in Supplementary Table S3, the estimated differences in GPES values were not significantly different between the two groups (*p* = 0.24).

Serious AEs were not observed in any study participant, and all AEs that presented during and after interventions were mild and transient (Supplementary Table S4). Hypotension requiring additional fluid or vasopressor administration was observed in three participants, who recovered immediately after conservative treatment. 

**Figure 4:**
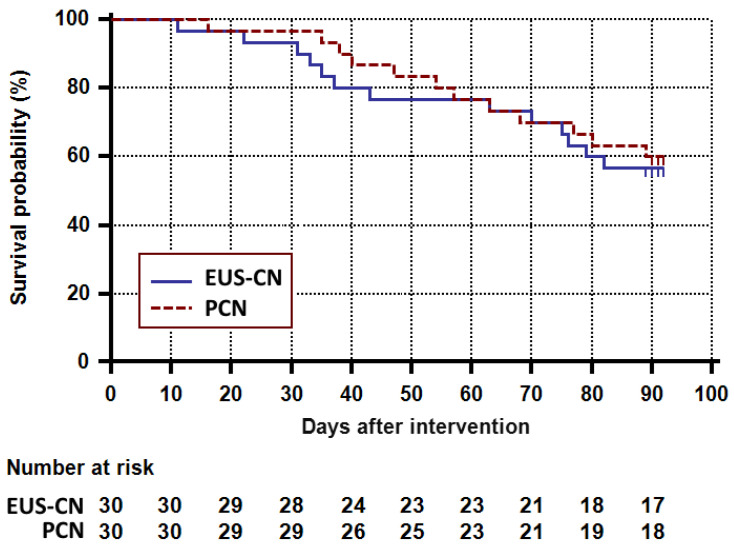
Survival analysis in patients with pancreatic cancer after EUS-CN or PCN. *p*-value for the Kaplan–Meier analysis of survival between the two groups was 0.73. EUS-CN, endoscopic ultrasound-guided celiac neurolysis; PCN, percutaneous celiac neurolysis.

Survival rates at 3 months in the EUS-CN group and the PCN group were 56.7% (95% CI: 37.3 to 72.1) and 60.0% (95% CI: 40.5 to 75.0), which did not show significant differences according to Kaplan–Meier analysis (*p* = 0.73; Figure 4). Finally, as shown in Supplementary Figure S3, subgroup analysis revealed that patients who underwent EUS-guided celiac ganglion neurolysis plus CPN (*n* = 25) had a higher survival rate than those who only underwent EUS-CPN (*n* = 5), albeit not statistically significant (*p* = 0.054).

## 4. Discussion

In this first-ever randomized, assessor-blind clinical trial comparing EUS-CN and PCN for pain management in cancer patients, the two methods showed comparable efficacy in relieving intractable pain in patients with pancreatic cancer and decreasing opioid consumption. Moreover, the two methods showed similar results in successful pain response, improvement of QOL, patient satisfaction, incidence of AEs, and survival rates at 3 months after intervention. 

PCN has been shown to be beneficial in the management of pain in cancer patients. A Cochrane review published in 2011 analyzed six papers on the efficacy of PCN in the management of pain in patients with pancreatic cancer [5] and showed that PCN was superior to systemic analgesic therapy in reducing pain scores and opioid consumptions. Another systematic review reported that PCN was beneficial for pain management by reducing opioid consumption and associated side effects in patients with abdominal cancer [12]. 

There has been no direct comparison of EUS-CN and PCN in the management of pain in patients with pancreatic cancer. It has been postulated that CN under EUS guidance might be a better method than PCN, as the echoendoscope is placed very close to the point where the celiac trunk emerges from the aorta, and the puncture of surrounding blood vessels can be avoided using Doppler imaging [5]. Another potential advantage of EUS-CN is that it can be performed alongside a diagnostic EUS examination [14]. However, in our study, the two methods were comparable in the degree of pain alleviation. We speculate that this may be due to anatomical characteristics (i.e., antecrural injection in EUS-CN vs. retrocrural injection in PCN) of the two methods; although EUS-CN has the aforementioned theoretical advantages, the antecrural injection method used in EUS-CN may result in faster dissipation of the injected alcohol in the celiac space, thus lowering the efficacy and durability than anticipated [30]. Indeed, a recent study showed that EUS-guided celiac ganglion radiofrequency ablation provided better pain relief and improved QOL when compared with EUS-guided chemical ablation of celiac plexus [31]; the authors of this study proposed that variations in the diffusion of the injected alcohol within the celiac space may have contributed to the relatively poor outcomes of EUS-guided chemical ablation. Another potential disadvantage of the antecrural injection is that tumors often infiltrate the antecrural space in advanced cases of pancreatic cancer, which inhibit adequate delivery of the neurolytic agent and compromise the efficacy of antecrural neurolysis [32]. We used the retrocrural injection method for PCN in our study; notably, it has been hypothesized that retrocrurally injected neurolytic agents spread periaortically, thus providing additional neurolytic effect to precrural components of the plexus [24]. In addition, we used CT simulation prior to PCN, which may have resulted in more accurate delivery of neurolytic agents than fluoroscopic or ultrasound guidance without simulation before procedure. Finally, the advantage of PCN over EUS-CN is that the approach can be either from the left or right side. On the other hand, EUS-CN has a disadvantage in that the drug tends to be distributed on the left side only. Because we did not implement a bilateral approach for EUS-CN, the results of EUS-CN here may be worse than previously reported.

This study has several strengths resulting from its assessor-blind design, which is crucial when participants and performing physicians cannot be blinded to the methods being used. In our study, the evaluator/assessor and the statistician were blind to the treatment allocation, and the performing physicians did not carry any active role in patient care following the interventions. Our study has some limitations of note. For sample size calculation, we used the NRS values (4.28 in EUS-CPN, 3.69 in percutaneous fluoroscopy-guided transdiscal CPN) reported in previous studies [12,29]. However, we observed lesser degrees of changes in the mean pain scores with EUS-CN and PCN. In this study, patients with advanced pancreatic cancer and intractable pain despite conventional opioid medication were enrolled. Therefore, the reduction of pain intensity in both methods of CN of this study may have been modest compared with previous studies that reported analgesic effect maintaining up to 3 months after the procedure [8]; this holds important clinical implications because lower opioid consumption would lead to fewer incidence of opioid-related AEs [12]. In addition, the procedures were performed at a late timing, after 30 weeks or more from the diagnosis. Because EUS-CN is performed using an anterior approach, it appears that tumor invasion may have had a greater impact on its therapeutic efficacy. As noted in a previous study [18], earlier timing for EUS-CN may result in a favorable outcome. Therefore, further randomized trial comparing EUS-CN and PCN may be required for this issue. Meanwhile, early identification and treatment of patients with intractable pain through multidisciplinary discussion would help to implement the effectiveness of EUS-CN. Also, the difference in the direction of neurolytic agent injection between the two methods hindered a more direct comparison; therefore, a future study may benefit from comparing EUS-CN and PCN that both use antecrural approaches. In addition, randomized controlled trials comparing novel EUS-CN methods such as radiofrequency ablation and PCN would be interesting. Moreover, the long-term efficacy of treatment could not be assessed because the last follow-up was scheduled for 3 months after intervention. Finally, cost analysis was not performed in this study. 

In conclusion, both EUS-CN and PCN may be regarded as viable options in the management of intractable pain in pancreatic cancer patients. Physicians may choose between EUS-CN and PCN depending on the level of technical expertise and availability of resources at each institution.

## Figures and Tables

**Table 1:** Patient demographics and clinical characteristics.

Variables	EUS-CN (*n* = 30)	PCN (*n* = 30)
Age, mean (SD), years	58.4 (9.6)	61.2 (7.4)
Male, No. (%)	15 (50.0)	20 (66.7)
Height, mean (SD), cm	162.4 (10.3)	162.8 (9.1)
Weight, mean (SD), kg	53.7 (8.6)	55.8 (9.2)
Comorbidity, No. (%)		
Diabetes	10 (33.3)	15 (50.0)
Hypertension	11 (36.7)	14 (46.7)
Pain duration, median (IQR), months	6.0 (2.0 to 10.0)	6.0 (4.0 to 11.0)
Pain intensity, median (IQR), NRS ^a^		
Abdominal pain	6.0 (4.0 to 8.0)	6.0 (4.0 to 8.0)
Back pain	6.0 (4.0 to 8.0)	6.0 (4.0 to 8.0)
Pain area, No. (%)		
Abdomen and back	7 (11.7)	14 (23.3)
Abdomen only	20 (33.3)	11 (36.7)
Back only	3 (10.0)	5 (16.7)
Pancreas cancer location No. (%)		
Head	15 (50.0)	17 (56.7)
Body and tail	19 (63.3)	13 (43.3)
Overall stage at intervention		
III	4 (13.3)	6 (20.0)
IV	26 (86.7)	24 (80.0)
MED, mean (SD), mg/day	221.7 (162.3)	214.2 (146.1)
BDI, mean (SD) ^b^	21.4 (10.0)	21.2 (10.1)
NFHSI, mean (SD) ^c^	35.3 (10.5)	36.5 (7.1)
Interval from diagnosis to CN, median (IQR), weeks	32.0 (21.0 to 54.0)	34.0 (20.0 to 50.0)

EUS-CN, endoscopic ultrasound-guided celiac neurolysis; PCN, percutaneous celiac neurolysis; NRS, numerical rating scale; SD, standard deviation; IQR, interquartile range; CN, celiac neurolysis; MED, total daily oral morphine equivalent dose; BDI, Beck depression inventory; NFHSI, National Comprehensive Cancer Network Functional Assessment of Cancer Therapy Hepatobiliary-Pancreatic Symptom Index. ^a^ NRS ranges from 0 (no pain) to 10 (worst pain possible). ^b^ BDI scores range from 0–63, with higher scores indicating more depressive mood. ^c^ NFHSI scores range from 0 to 72, with higher scores indicating more decreased quality of life.

**Table 2:** Pain scores after EUS-CN or PCN.

Variables	Time	Adjusted Prediction (95% CI) ^a^		Estimated Difference (95% CI) ^b^	*p*-Value
		EUS-CN	PCN		
Abdominal pain	Baseline	6.0 (5.1 to 7.0)	5.5 (4.6 to 6.5)	−0.5 (−1.8 to 0.8)	0.46
(NRS)	1 week	4.8 (3.9 to 5.8)	3.8 (2.9 to 4.8)	−1.0 (−2.3 to 0.3)	0.15
	2 weeks	4.6 (3.6 to 5.5)	3.9 (2.9 to 4.8)	−0.7 (−2.0 to 0.7)	0.33
	1 month	4.7 (3.7 to 5.7)	3.7 (2.7 to 4.6)	−1.0 (−2.4 to 0.4)	0.17
	2 months	5.4 (4.3 to 6.5)	3.9 (2.9 to 5.0)	−1.5 (−3.0 to 0.0)	0.052
	3 months	5.6 (4.4 to 6.9)	4.2 (3.0 to 5.4)	−1.4 (−3.1 to 0.3)	0.11
Back pain	Baseline	5.5 (4.5 to 6.5)	5.6 (4.7 to 6.6)	0.1 (−1.2 to 1.5)	0.85
(NRS)	1 week	3.7 (2.7 to 4.7)	3.1 (2.1 to 4.0)	−0.7 (−2.0 to 0.7)	0.35
	2 weeks	4.0 (3.0 to 5.0)	3.0 (2.0 to 4.0)	−1.0 (−2.4 to 0.4)	0.17
	1 month	3.6 (2.6 to 4.6)	2.7 (1.7 to 3.7)	−0.9 (−2.4 to 0.5)	0.21
	2 months	4.3 (3.2 to 5.4)	3.4 (2.3 to 4.4)	−0.9 (−2.5 to 0.6)	0.24
	3 months	4.3 (3.1 to 5.6)	3.4 (2.2 to 4.6)	−0.9 (−2.7 to 0.9)	0.31

CI, confidence interval; EUS-CN, endoscopic ultrasound-guided celiac neurolysis; PCN, percutaneous celiac neurolysis; NRS, numerical rating scale. **^a^** A linear mixed model was used for statistical analysis. ^b^ Estimated difference in values between the two groups at each time.

**Table 3:** Successful pain response after EUS-CN or PCN.

Variable	Time	Estimated Proportion (95% CI) ^b^		Difference of Proportion (95% CI) ^c^	*p*-Value ^d^
		EUS-CN	PCN		
Successful	1 week	60.0 (42.5 to 77.5)	56.7 (38.9 to 74.4)	−3.3 (−28.5 to 21.8)	0.79
Responder ^a^	2 weeks	46.7 (28.8 to 64.5)	53.3 (35.5 to 71.2)	6.7 (−18.8 to 32.2)	0.61
	1 month	56.7 (38.9 to 74.4)	70.0 (53.6 to 86.4)	13.3 (−11.3 to 37.9)	0.29
	2 months	30.0 (13.6 to 46.4)	43.3 (25.6 to 61.1)	13.3 (−11.3 to 37.9)	0.29
	3 months	23.3 (8.2 to 38.5)	36.7 (19.4 to 53.9)	14.6 (−9.1 to 38.3)	0.26

Data are expressed as estimated proportions (%) and 95% confidence interval (CI). EUS-CN, endoscopic ultrasound-guided celiac neurolysis; PCN, percutaneous celiac neurolysis. ^a^ Successful response was defined as a decrease in numerical rating scale ≥50% relative to or ≥3-point reduction from baseline. **^b^** A generalized estimating equation was used in the statistical analysis. ^c^ Difference of proportion between the two groups at each time. ^d^ Overall *p*-value between the two groups for successful responders = 0.33.

## Supplementary Materials

The following are available online at https://www.mdpi.com/2077-0383/9/6/1666/s1. Methods. Supplementary Figure S1. Adjusted predictions of pain scores after endoscopic ultrasound-guided celiac neurolysis or percutaneous celiac neurolysis. Supplementary Figure S2. Adjusted predictions of quality of life after endoscopic ultrasound-guided celiac neurolysis or percutaneous celiac neurolysis. Supplementary Figure S3. Kaplan–Meier analysis of patient survival following endoscopic ultrasound-guided celiac ganglion neurolysis plus celiac plexus neurolysis or celiac plexus neurolysis only. Supplementary Table S1. Baseline characteristics of the type and amounts of opioids. Supplementary Table S2. Adjusted predictions of quality of life after endoscopic ultrasound-guided celiac neurolysis or percutaneous celiac neurolysis. Supplementary Table S3. Adjusted predictions of the global perceived effect of satisfaction after endoscopic ultrasound-guided celiac neurolysis or percutaneous celiac neurolysis. Supplementary Table S4. Cumulative rates of the complications following endoscopic ultrasound-guided celiac neurolysis or percutaneous celiac neurolysis.
