# Supplementary material for: EUS-Guided Versus Percutaneous Celiac Neurolysis for the Management of Intractable Pain Due to Unresectable Pancreatic Cancer: A Randomized Clinical Trial
Source: J Clin Med. 2020 Jun 1;9(6):1666. doi: 10.3390/jcm9061666 (PMC7356927; doi:10.3390/jcm9061666)
Supplement: Supplementary file 1 [file jcm-09-01666-s001.pdf]

Supplementary Material

Methods

Supplementary Figure 1. Adjusted predictions of pain scores after endoscopic ultrasound-guided celiac neurolysis or percutaneous celiac neurolysis

Supplementary Figure 2. Adjusted predictions of quality of life after endoscopic ultrasound-guided celiac neurolysis or percutaneous celiac neurolysis

Supplementary Figure 3. Kaplan-Meier analysis of patient survival following endoscopic ultrasound-guided celiac ganglion neurolysis plus celiac plexus neurolysis or celiac plexus neurolysis only

Supplementary Table 1. Baseline characteristics of the type and amounts of opioids

Supplementary Table 2. Adjusted predictions of quality of life after endoscopic ultrasound-guided celiac neurolysis or percutaneous celiac neurolysis

Supplementary Table 3. Adjusted predictions of the global perceived effect of satisfaction after endoscopic ultrasound-guided celiac neurolysis or percutaneous celiac neurolysis

Supplementary Table 4. Cumulative rates of the complications following endoscopic ultrasound-guided celiac neurolysis or percutaneous celiac neurolysis

Methods

Endoscopic ultrasound-guided celiac neurolysis (EUS-CN)

EUS-CNs were carried out on an inpatient basis. Prophylactic antibiotics were administered before the procedure. After placing the patients in a left lateral position, the procedures were performed under moderate or deep sedation as previously described [1]. Briefly, intravenous midazolam (0.05 mg/kg body weight or 1 mg if age > 70 or ASA class III) and 25 mg of meperidine (12.5 mg if age > 70 or ASA class III) were administered; repeated doses of 10-20 mg of propofol were intravenously administered to maintain moderate or deep sedation. The site of celiac artery take-off from the aorta was identified using a curvilinear echoendoscope (GF-UCT 260, Olympus Medical Systems, Tokyo, Japan). The tip of a 22-gauge EUS needle (EchoTip® Ultra, Cook Medical, Winston-Salem, NC, USA) was introduced to the celiac plexus area or the celiac ganglia under EUS guidance. After confirming that the needle tip was not intravascular using Doppler and aspiration, 10 mL of 0.5% bupivacaine was injected, followed by 15 mL of 99.5% dehydrated alcohol [2]. When the celiac ganglia were identified, celiac ganglia neurolysis plus unilateral celiac plexus neurolysis was performed; when the celiac ganglia were not identified, unilateral celiac plexus neurolysis was performed.

The patients were monitored in the recovery room for 30 minutes after the completion of the procedure. Unless contraindicated, 500 mL of normal saline was

intravenously administered during the recovery period. The patients were then transferred to the ward.

Percutaneous celiac neurolysis (PCN)

For PCNs, we utilized fluoroscopy-guided transdiscal approach [3]. Before the procedure, simulation for the transdiscal needle pathway was performed using the most recent abdominal computed tomography (CT) images. Briefly, the pathway was drawn from the midpoint of the anterior border of T12-L1 intervertebral disc to the lateral side of the right superior articular process of the T12 vertebra and extended to the skin on the axial CT image. The point at which the pathway crossed the posterior skin surface was planned as the needle insertion point. Also, the needle insertion angle between the proposed needle pathway and the midline was measured for fluoroscopy-guidance [4].

Premedication or sedatives were not used for PCN. Blood pressure, electrocardiogram, and oxygen saturation of the patients were monitored during the procedure. A fluoroscopy C-arm system (OEC 9800, General Electric Healthcare, Little Chalfont, United Kingdom) was used. In the operating room, patients were placed in a prone position. After identifying the T12-L1 intervertebral disc space by fluoroscopic anteroposterior imaging, the C-arm was adjusted to superimpose the T12-L1 endplates. The fluoroscopic C-arm was rotated to the right side of the patient to the insertion angle predetermined by CT simulation. The insertion point was the lateral margin of the superior articular process of the T12. After skin preparation,

both the skin and soft tissues were injected with 1% lidocaine. The skin was punctured with an 18-gauge needle 38 mm in length, and a 22-gauge Chiba needle (GMS, Hwaseong-si, Korea) was introduced through the 18-gauge needle. After contacting the disc, the fluoroscope was rotated to the lateral position. The needle was advanced through the disc while the tip position was frequently checked with the AP and lateral fluoroscopic images. Immediately after penetrating the disc, the position of the needle was confirmed on anteroposterior and lateral views by injecting a 10 ml-mixture containing 5 mL of contrast medium (Omnipaque, Nycomed Imaging AS, Oslo, Norway) and 5 mL of 2% lidocaine to identify the spreading pattern and to prevent pain from the injection of alcohol. If there were no obvious adverse effects after 3 minutes, 10 ml of 99.5% dehydrated alcohol was injected for neurolysis.

The patients were monitored in the recovery room for 30 minutes and maintained the prone position for 2 hours to prevent unwanted spread of the neurolytic agent to adjacent structures.

REFERENCES

1. Paik W.H.; Lee T.H.; Park D.H.; Choi J.H.; Kim S.O.; Jang S.; Kim D.U.; Shim J.H.; Song T.J.; Lee S.S.; et al. EUS-guided biliary drainage versus ERCP for the primary palliation of malignant biliary obstruction: a multicenter randomized clinical trial. *Am. J. Gastroenterol.* **2018**, *113*, 987-997, doi: 10.1038/s41395-018-0122-8.

2. Kappelle, W.F.W.; Bleys, R.; van Wijck, A.J.M.; Siersema, P.D.; Vleggaar, F.P. EUS-guided celiac ganglia neurolysis: a clinical and human cadaver study (with video). *Gastrointest. Endosc.* **2017**, *86*, 655-663, doi:10.1016/j.gie.2017.01.041.
3. Yamamuro, M.; Kusaka, K.; Kato, M.; Takahashi, M. Celiac plexus block in cancer pain management. *Tohoku J. Exp. Med.* **2000**, *192*, 1-18.
4. Kong, Y.G.; Shin, J.W.; Leem, J.G.; Suh, J.H. Computed tomography (CT) simulated fluoroscopy-guided transdiscal approach in transcrural celiac plexus block. *Korean J. Pain* **2013**, *26*, 396-400, doi:10.3344/kjp.2013.26.4.396.

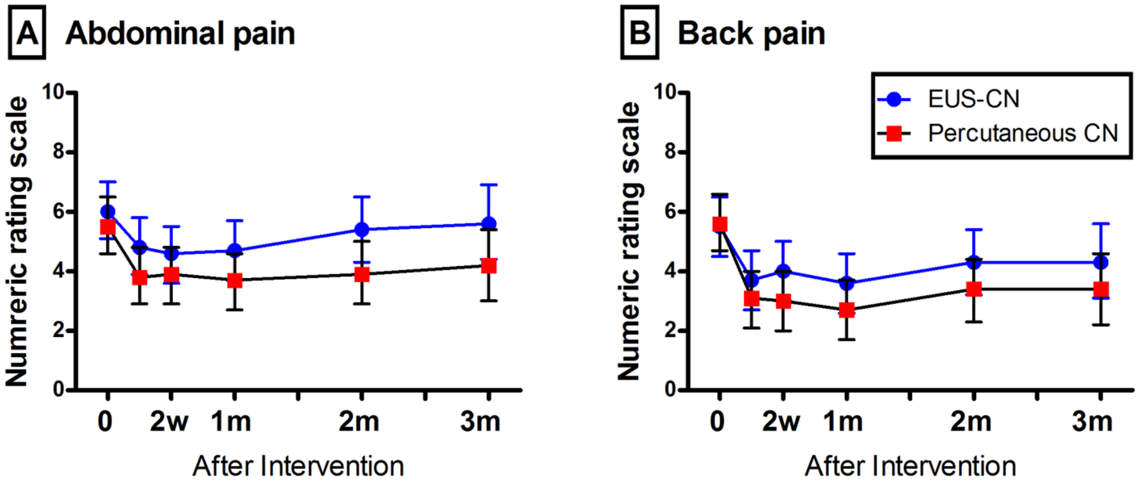

Supplementary Figure 1. Adjusted predictions of pain scores after endoscopic ultrasound-guided celiac neurolysis or percutaneous celiac neurolysis. A linear mixed model was used for statistical analysis. Overall *P* values between two groups for abdominal and back pain were 0.048 and 0.152, respectively. EUS-CN, endoscopic ultrasound-guided celiac neurolysis; CN, celiac neurolysis.

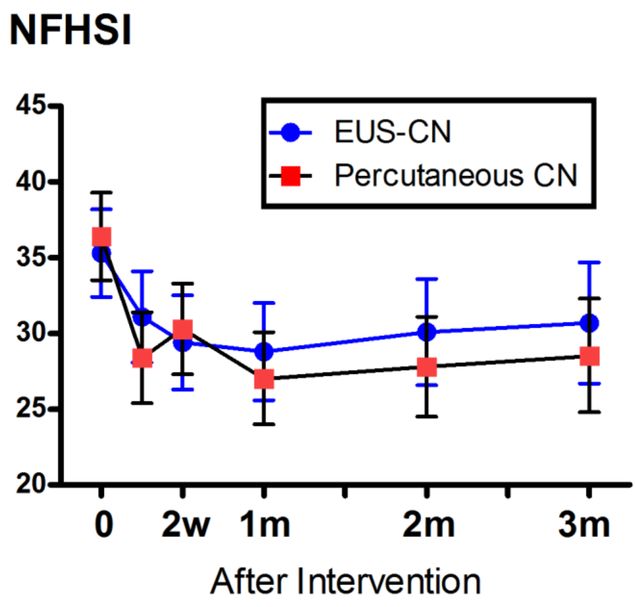

Supplementary Figure 2. Adjusted predictions of quality of life after endoscopic ultrasound-guided celiac neurolysis or percutaneous celiac neurolysis. A linear mixed model was used for statistical analysis. Overall *P* value between the two groups for NFHSI = 0.440. EUS-CN, endoscopic ultrasound-guided celiac neurolysis; CN, celiac neurolysis; NFHSI, National Comprehensive Cancer Network Functional Assessment of Cancer Therapy Hepatobiliary-Pancreatic Symptom Index.

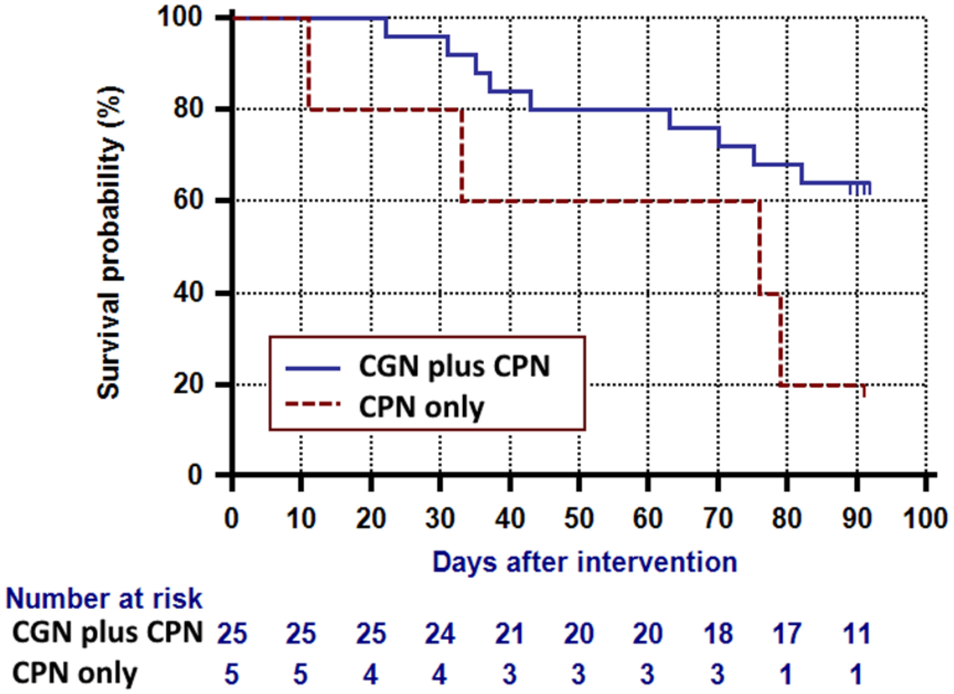

Supplementary Figure 3. Kaplan-Meier analysis of patient survival following endoscopic ultrasound-guided celiac ganglion neurolysis plus celiac plexus neurolysis or celiac plexus neurolysis only. *P* value for comparison between participants who underwent EUS-CGN plus CPN (*n* = 25) and those who underwent EUS-CPN only (*n* = 5) = 0.054. CGN, celiac ganglion neurolysis; CPN, celiac plexus neurolysis; EUS, endoscopic ultrasound.

Supplementary Table 1. Baseline characteristics of the type and amounts of opioids.

Variables	EUS-CN (n=30)	PCN (n=30)	*p* value
**Oxycodone (mg), oral**	25.0 (15.0–45.0)	13.75 (10.0–50.0)	0.23
**Oxycodone (mg), iv**	39.0 (20.0–58.0)	40.0 (30.0–92.0)	0.56
**Oxycodone with naloxone (mg), oral**	50.0 (40.0–80.0)	60.0 (20.0–80.0)	0.81
**Morphine (mg), iv**	40.0 (15.0–50.0)	44.0 (24.070.0)	0.31
**Tapentadol (mg), oral**	250.0 (250.0250.0)	200.0 (200.0–200.0)	0.16
**Hydromorphone (mg), iv**	16.0 (16.0–16.0)	0	N/A
**Pethidine (mg), iv**	0	37.5 (25.0–62.5)	N/A
**Fentanyl (mcg), transdermal**	25.0 (25.075.0)	25.0 (12.5–50.0)	0.51
**Fentanyl (mcg), iv**	400.0 (200.0–400.0)	250.0 (200.0–450.0)	0.71

EUS-CN, endoscopic ultrasound-guided celiac neurolysis; PCN, percutaneous celiac neurolysis; N/A, not applicable.

Supplementary Table 2. Adjusted predictions of quality of life after endoscopic ultrasound-guided celiac neurolysis or percutaneous celiac neurolysis.

Variables	Time	Adjusted prediction (95% CI)[a]		Estimated difference (95% CI)[b]	*p* value[c]
		EUS-CN	PCN		
NFHSI	Baseline	35.3 (32.4–38.2)	36.4 (33.5–39.3)	1.1 (−3.1 to 5.2)	0.61
	1 week	31.1 (28.1–34.1)	28.4 (25.4–31.4)	−2.7 (−6.9 to 1.5)	0.21
	2 weeks	29.4 (26.3–32.5)	30.3 (27.3–33.3)	0.9 (−3.5 to 5.2)	0.70
	1 month	28.8 (25.6–32.0)	27.0 (24.0–30.1)	−1.8 (−6.2 to 2.6)	0.43
	2 months	30.1 (26.6–33.6)	27.8 (24.5–31.1)	−2.3 (−7.1 to 2.5)	0.34
	3 months	30.7 (26.7–34.7)	28.5 (24.8–32.3)	−2.2 (−7.6 to 3.3)	0.43

CI, confidence interval; EUS-CN, endoscopic ultrasound-guided celiac neurolysis; PCN, percutaneous celiac neurolysis; NFHSI, The National Comprehensive Cancer Network Functional Assessment of Cancer Therapy Hepatobiliary-Pancreatic Symptom Index. [a]A linear mixed model was used for statistical analysis. [b]Estimated difference in values between groups at each time. [c]Overall *p* value between the two groups for NFHSI = 0.44.

Supplementary Table 3. Adjusted predictions of the global perceived effect of satisfaction after endoscopic ultrasound-guided celiac neurolysis or percutaneous celiac neurolysis.

Variable	Time	Adjusted prediction (95% CI)[b]		Estimated difference (95% CI)[c]	*p* value[d]
		EUS-CN	PCN		
GPES[a]	1 week	4.4 (3.5–5.3)	4.8 (3.9–5.6)	0.3 (−0.9 to 1.6)	0.59
	2 weeks	4.4 (3.4–5.3)	4.7 (3.8–5.6)	0.3 (−1.0 to 1.6)	0.63
	1 month	3.9 (2.9–4.8)	4.7 (3.8–5.6)	0.9 (−0.5 to 2.2)	0.20
	2 months	5.2 (4.1–6.2)	4.9 (3.9–5.9)	−0.3 (−1.7 to 1.2)	0.73
	3 months	3.7 (2.5–5.0)	4.9 (3.7–6.0)	1.2 (−0.5 to 2.8)	0.18

CI, confidence interval. EUS-CN, endoscopic ultrasound-guided celiac neurolysis; PCN, percutaneous celiac neurolysis; GPES, global perceived effect of satisfaction. [a]GPES scores range from 1–7, with higher scores indicating higher satisfaction. [b]A linear mixed model was used for statistical analysis. [c]Estimated difference in values between the two groups at each timepoint. [d]Overall *p* value between the two groups for GPES = 0.24.

Supplementary Table 4. Cumulative rates of the complications following endoscopic ultrasound-guided celiac neurolysis or percutaneous celiac neurolysis.

Variables	EUS-CN (n=30)	PCN (n=30)	*P* value
Overall complications	11 (36.7)	10 (33.3)	>0.99
Procedure-related pain	8 (26.7)	4 (13.3)	0.33
Diarrhea	4 (13.3)	4 (13.3)	>0.99
Hypotension	1 (3.3)	2 (6.7)	>0.99

EUS-CN, endoscopic ultrasound-guided celiac neurolysis; PCN, percutaneous celiac neurolysis.
